# De Novo CD5+ Primary Gastrointestinal Diffuse Large B-Cell Lymphoma: Challenges With Treatment and Clinical Course

**DOI:** 10.1177/2324709619893546

**Published:** 2019-12-07

**Authors:** Preethi Ramachandran, Sonu Sahni, Jen C. Wang

**Affiliations:** 1 Brookdale University Hospital and Medical Center, Brooklyn, NY, USA

**Keywords:** DLBCL, gastrointestinal lymphoma, CD5+ lymphoma, B-cell lymphoma, small bowel lymphoma

## Abstract

The gastrointestinal tract is a common extranodal site for lymphomas. However, primary gastrointestinal lymphomas are rare. Diffuse large B-cell lymphomas (DLBCL) are the most commonly encountered type in the gastrointestinal tract. Most of the DLBCL are CD5 negative. CD5+ DLBCL is very rare and a poor prognostic subtype of lymphoma. We report a rare case of primary small bowel CD5+ DLBCL that evolved from being a localized low International Prognostic Index–scored disease into an advanced and aggressive disease primarily dictated by the presence of CD5 antigen positivity.

## Background

Primary gastrointestinal (GI) lymphoma is a very rare entity, constituting only about 1% to 4% of all GI malignancies.^1^ Diffuse large B-cell lymphoma (DLBCL) represents the most common histological subtype of primary GI lymphoma and is a heterogeneous group of disease.^2^ DLBCL constitutes about 40% of non-Hodgkin’s lymphomas.^3^ Although lymphoma can involve any part of the GI tract, the most common sites in order of its occurrence are the stomach followed by small intestine and ileocecal region.^4^ De novo CD5+ DLBCL in the small intestine is a very rare phenomenon, and so far, only 2 cases have been reported in the literature. CD5 expression in DLBCL is a poor prognostic marker. CD5 is usually expressed in chronic lymphocytic leukemia, mantle cell lymphoma (MCL), and less frequently in DLBCL. De novo CD5+ DLBCL is a rare and poor prognostic subtype of lymphoma. They account of at least 10% of all DLBCLs.^5,6^ DLBCL-expressing CD5 antigen seems to be a unique subgroup that is phenotypically, genotypically, and immunophenotypically different from the other CD5− DLBCLs. This rare immunophenotypic lymphoma has clinical and prognostic implications. We describe a case of de novo CD5+ DLBCL involving small intestine, which differed from all the clinical characteristics previously studied for this subtype.

## Case Description

**Figure 1.**
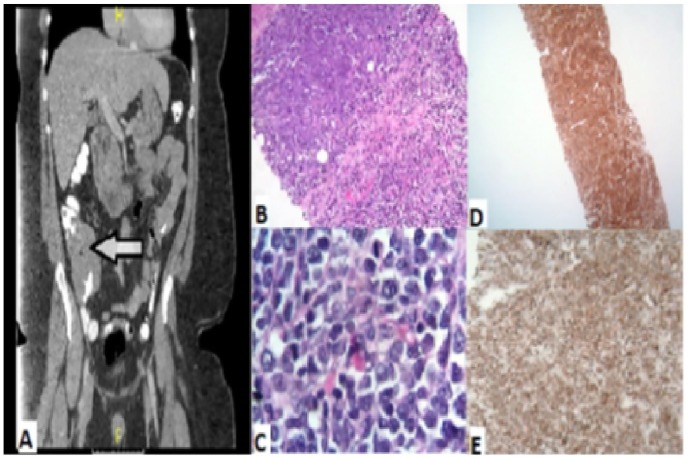
Imaging and pathology of the initial CD5+ small bowel diffuse large B-cell lymphoma. (A) Computed tomography scan showing isolated small bowel mass. (B and C) Hematoxylin and eosin, original magnification ×4 (B), ×100 (C), showing large centroblastic lymphocytes. (D) CD5, original magnification ×4. (E) CD79a, original magnification ×50.

**Figure 2.**
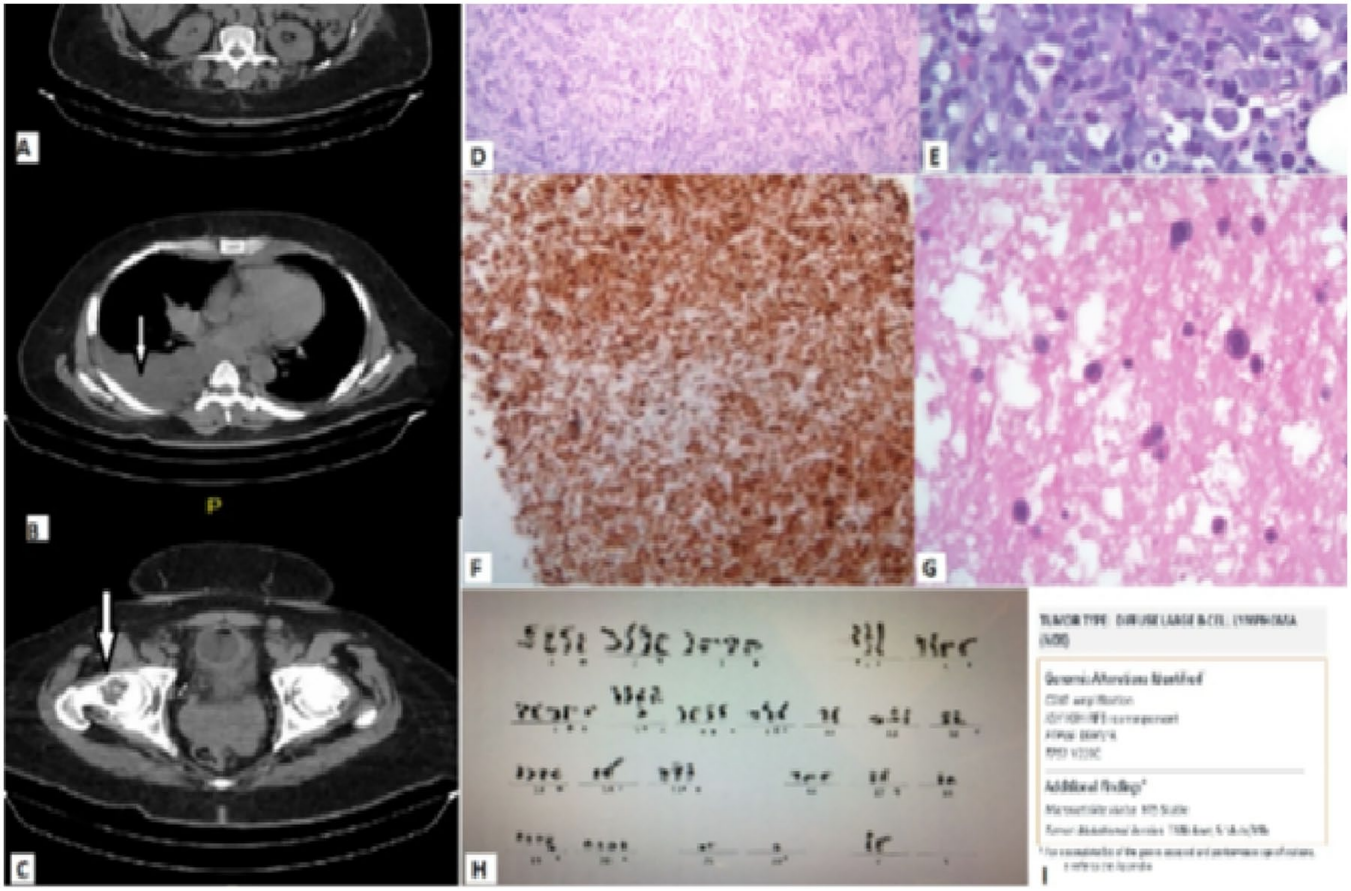
Imaging, pathology, and genetic analysis of the relapsed CD5+ diffuse large B-cell lymphoma. (A) Omental caking in abdomen. (B) Right-sided pleural effusion with thoracic lymphadenopathy. (C) Bony lytic lesion in the right femur. (D and E) Hematoxylin and eosin, original magnification ×4 (D), ×100 (E), showing large centroblastic lymphocytes. (F) CD5, (G) pleural fluid—hematoxylin and eosin, original magnification ×50, demonstrating large centroblastic lymphocytes in pleural fluid. (H) Cytogenetic analysis showing complex karyotyping. (I) Genetic sequencing analysis.

This is a 50-year-old gentleman who presented with symptomatic anemia secondary to severe iron deficiency. On imaging, he was found to have an exophytic soft tissue mass in the cecal tip adjacent to appendix. His colonoscopy was consistent with a near circumferential large polypoid mass at the ileocecal valve extending into the terminal ileum. Biopsy of the ileocecal mass (Figure 1) showed diffuse infiltrate of large (centroblast-like) lymphoid cells positive for CD20, CD5, CD23, CD43, BCL-2, BCL-6, c-MYC, MUM-1, and with Ki-67 of 60%. Fluorescence in situ hybridization did not show any evidence for MYC, CCND1-IGH, BCL2-IGH, BCL-6 rearrangements. The immunohistochemical and flow cytometry analysis resembled Richter syndrome although chronic lymphocytic leukemia was not preexisting. MCL was ruled out by absence of cyclin-D1 by fluorescence in situ hybridization analysis. His bone marrow biopsy was negative for any involvement of lymphoma and he did not have any evidence of lymphadenopathy or splenomegaly on imaging. His final diagnosis was consistent with stage I, primary GI de novo CD5+ DLBCL of activated B-cell type with low to intermediate IPI (International Prognostic Index) score. After reviewing the literature, and discussions in the tumor board, we opted for the initial surgery followed by chemotherapy. He successfully underwent laparoscopic right colectomy without any postsurgical complications. But within few weeks of being treated with chemotherapy, he presented with new right-sided pleural effusion, diffuse lymphadenopathy, peritoneal carcinomatosis, bony lytic lesions, new thromboembolism, and tumor lysis syndrome. Biopsy of the omental mass (Figure 2) confirmed progression of CD5+ DLBCL. His cytogenetic analysis showed complex karyotype, and next-generation sequence analysis showed TP53 mutation, a poor prognostic marker. His lymphoma was very aggressive causing multi-organ damage, making it very difficult for salvage therapy. Finally, he died within few days due to cardiorespiratory failure.^6^

## Discussion

The most common extranodal site for lymphoma is the GI tract.^1^ On the other hand, primary GI lymphoma are very rare. It accounts for about 1% to 4% of GI malignancies.

Stomach is the most common involved sites than intestine and ileocecal area.^2^ Primary GI lymphoma is usually of B-cell lineage, with certain histological subtypes having a relative predilection sites. For instance, MALT (mucosa-associated lymphoid tissue) lymphoma is common in stomach, whereas MCL in terminal ileum, jejunum and colon, and follicular lymphoma is usually found in the duodenum.^4^ Since 1961, Dawson’s criteria^7^ have been used to characterize primary GI lymphomas; these are negative peripheral lymphadenopathy, negative mediastinal lymphadenopathy, normal white blood cell counts, predominance of bowel lesion with only lymph nodes affected in the immediate vicinity, and no liver or spleen involvement. Molecular studies have shown that CD5 is a negative regulator of B-cell receptor signaling,^8^ modifies intracellular calcium, and modulates B-cell physiology by activating various signaling pathways, including ERK1/2, PI3K, and calcineurin.^9^ CD5 also adds to B-cells survival advantage through stimulation of autocrine interluekin-10 production.^10^ The mechanism leading to overexpression of CD5 in DLBCL remains unclear. The majority of CD5+ DLBCL cases belong to the activated B-cell subtype of DLBCL.^11,12^ Clinically, this molecular subtype of DLBCL follows an aggressive clinical course similar to T-cell lymphomas.^13^

The role of surgical intervention in primary colon lymphoma was reviewed by Cai et al.^14^ Different treatment approaches have been studied previously involving surgery plus chemotherapy and chemotherapy alone for localized GI lymphomas. Early tumor stage, right-sided lesion, and DLBCL histological pattern seem to be the clinical characteristics of optimal surgical candidates.^15^ For patients with localized disease (Lugano stage I/II), surgery plus chemotherapy yielded a lower relapse rate (15.3%) than did chemotherapy alone (36.8%, *P* < .001). The 3-year overall survival (OS) rate was 91% in the surgery plus chemotherapy group and 62% in the chemotherapy-alone group.^16^ By opting for the surgical approach, we reduced his risk of perforation during therapy as well as his exposure to chemotherapy side effects by reducing the number of chemotherapy cycles.

Although CD5− DLBCL commonly involves GI tract, de novo CD5+ DLBCL involvement of GI tract is very uncommon. An extensive case study was done by Harada et al^5^ analyzing the clinical characteristics of 63 cases of DLBCL by grouping them as CD5+, CD5− CD10+, and CD5− CD10−. As per the study, the median age at diagnosis was 57 years, with slightly more preponderance in females. Sixty-two percent of the cases had advanced stages (III and IV), and almost half of the patients had extranodal involvement.^5,17^ The extranodal sites included arm, nasal cavity, skin, spleen, stomach, testis, tonsil, thyroid, breast, and GI tract with highest number of cases in breast. Rare cases of involvement of liver has also been reported.^18^ Almost all of the patients did not show positivity for HIV or HTLV1. Ten percent of the cases showed BCL2 rearrangements, while 25% of the cases had BCL6 rearrangement. All cases had negative cyclin D1 by immunohistochemistry. As per this study, the clinical characteristics of this subgroup of CD5+ DLBCL included female preponderance, elderly onset, advanced stage at presentation, and with frequent involvement of bone marrow.^17^

Kobayashi et al published another larger study involving 109 de novo CD5+ DLBCL cases primarily studying their clinical characteristics.^19^ These cases were compared with 384 CD5− DLBCL and 128 cyclin D1–positive MCL cases. As per this study, CD5+ DLBCL patients had a higher age distribution with a median age of 66 years with a higher female preponderance. When compared with CD5-neagative DLBCL, the subgroup of patients with CD5 positivity showed aggressive clinical features, >1 performance status, higher lactate dehydrogenase (LDH), B symptoms, advanced stage at diagnosis, higher IPI, and with the involvement of more than one extranodal sites with bone marrow involvement being more frequent. Most of the CD5+ DLBCL subtypes showed centroblastic morphology and had immunophenotypic characteristics of CD5+, CD10−, CD19+, CD20+, CD23−, and cyclin D1−. The OS curve was significantly (*P* = .0026) inferior compared with CD5+ DLBCL.^19^

As per these large studies CD5 expression in DLBCL is associated with elevated LDH, B symptoms, extranodal involvement, poor performance status, higher IPI,^20^ and advanced stage at diagnosis with more frequent central nervous system involvement.^21^ But our patient had low LDH, no B symptoms, good performance status, low IPI score, and with localized stage at diagnosis thereby differing from the ones described in the studies. Although rituximab-based chemotherapy has improved the OS in CD5+ DLBCL, it still remains much lower compared with CD5− DLBCL. Unfortunately, the rate of central nervous system involvement has not improved with the use of rituximab.^21,22^

Even though we opted for the best treatment approach of upfront surgery followed by R-CHOP chemotherapy for the localized small bowel lymphoma to reduce the toxicity and improve the OS, the CD5 expression of the lymphoma dictated the prognosis irrespective of the mode of therapy. The disease showed an aggressive clinical course and progressed through the R-CHOP chemotherapy, proving the fact that these subtypes of lymphomas are molecularly and clinically different from CD5− DLBCL. Whether more intensive chemotherapy like REPOCH or Hyper-CVAD would have benefitted for these rare lymphomas is still a question of debate as there are no consensus in the treatment regimens for these unique subtypes.

## Conclusion

Our case posed a diagnostic challenge by presenting as a rare and unique immunotypic subtype of DLBCL in a unique location with poor prognostic features. Optimal management in these cases are not clear. Our case was very unique in presenting initially with good prognostic markers and later evolving into an aggressive variant with poor prognostic features, likely due to CD5 expression. Whether highly intensive regimens other than R-CHOP or even stem cell transplant would salvage these patients remains to be studied yet.
